# Bibliometric analysis of global research in palliative care for cervical cancer

**DOI:** 10.3389/fonc.2024.1432805

**Published:** 2024-08-26

**Authors:** Fhaied Almobarak

**Affiliations:** Fundamentals of Nursing Department, College of Nursing, Imam Abdulrahman Bin Faisal University, Dammam, Saudi Arabia

**Keywords:** cervical cancer, palliative care, Scopus, bibliometric analysis, Bibliometrix R Package’s web app Biblioshiny, VOSviewer

## Abstract

**Objective:**

The present study aims to conduct a comprehensive bibliometric analysis of global research in palliative care for cervical cancer, providing insights into publication trends, authorship patterns, influential journals, and thematic concentrations.

**Methods:**

A bibliometric analysis approach was employed using metadata extracted from Scopus spanning 2000-2023. The search utilized main terms related to cervical cancer and palliative care. Data analysis and visualization were performed using the Bibliometrix R Package’s web app Biblioshiny and VOSviewer software.

**Results:**

The study identified 2,492 publications on palliative care for cervical cancer, with a notable peak in 2021. The analysis revealed a diverse publication landscape, encompassing primarily articles. Citation analysis showed a staggering 63,994 citations. The most relevant journals were The Lancet Oncology, Gynecologic Oncology, and International Journal of Gynecological Cancer. The study also highlighted influential authors, institutions, and countries, with Harvard University, the University of Toronto, and the University of Texas MD Anderson Cancer Center leading in publications.

**Discussion:**

The findings reflected a growing interest in palliative care for cervical cancer, marked by increasing publications over the years. However, the analysis indicated limited international collaborations, with research efforts concentrated in high-income countries. Thematic areas include surgery, palliative care, chemotherapy, radiotherapy, and quality of life. Thus, further collaborations and research in developing countries are needed.

**Conclusion:**

This bibliometric analysis showcased a comprehensive overview of the global research landscape on palliative care for cervical cancer. The study identified trends, key contributors, and thematic concentrations, offering valuable insights for future research directions and enhancing palliative care services. Addressing the identified gaps, fostering international collaborations, and directing research efforts toward developing countries can contribute to the advancement of palliative care for cervical cancer globally.

## 1 Introduction

Cervical cancer remains a significant global health concern, ranking as the fourth most prevalent cancer among women worldwide. In 2022 alone, there were approximately 660,000 new cases and 350,000 deaths attributed to cervical cancer, and 94% of these deaths occurred in low and middle-income Human Development Index (HDI) countries (1). Despite initiatives such as the World Health Organization’s (WHO) strategy to eradicate cervical cancer through a triple-pillar approach: HPV vaccination, screening, and treatment, the impact of this disease persists (2). Innovative methods, such as single-dose vaccination, HPV DNA tests, and same-day screen-and-treat procedures, are being explored to improve prevention and treatment outcomes (3). It is anticipated that the successful implementation of these strategies could significantly reduce new cervical cancer cases and related deaths by 2050 (4). However, challenges remain in achieving the WHO’s cervical cancer elimination targets, predominantly in low and middle-income countries where the majority of the total cervical cancer cases and deaths occur (4). While preventive measures such as human papillomavirus (HPV) vaccination and cervical screenings have shown progress in reducing incidence rates, particularly in regions with high HIV prevalence, many women still confront challenges associated with advanced or metastatic cervical cancer and its mortality rates (5, 6).

Palliative care is a specialized approach that provides holistic support to patients facing life-limiting illnesses, focusing on pain and symptom management, communication, quality of life, family support, and grief support (7, 8). While traditionally associated with cancer care, the need for palliative care extends to all serious illnesses, emphasizing the importance of addressing suffering and preserving the welfare of the patients (9, 10). Palliative care is essential in cervical cancer management due to the high prevalence of late-stage diagnoses and significant symptom burden (11). Palliative care is particularly vital given that advanced cervical cancer can lead to severe symptoms that significantly interfere with quality of life (12). It addresses physical, psychological, social, and spiritual issues throughout the disease trajectory, improving patient satisfaction, treatment compliance, and well-being (11). Palliative care is needed to manage pain, bleeding, fistulas, and other complications from treatments such as radiation and chemotherapy, which can affect ovarian, urinary, and bowel function (13). An essential package of palliative care for cervical cancer (EPPCCC) is designed to be universally accessible, but some patients may require an augmented package to address refractory suffering, including specialized pain management, surgical procedures, and psycho-oncologic therapies (14). In short, the severity of physical, psychological, social, and spiritual suffering experienced by cervical cancer patients and their caregivers highlights the urgent need for global and regional strategic planning to align health investments with the identified needs for palliative care services (12, 15).

Bibliometric analysis uses mathematical and statistical methods to quantitatively analyze scientific literature (such as books, journal articles, and data sets, along with their associated metadata like abstracts, keywords, and citations), identifying patterns, trends, and impacts within specific fields (16). It involves data collection, cleaning, and subjecting data to various bibliometric methods to generate meaningful information. This analysis technique is increasingly popular for examining and assessing enormous amounts of scientific data to rectify flawed content or ethical concerns (17), structure and centralize literature efficiently, and provide researchers with trends and insights (18). Additionally, bibliometric analysis is crucial in understanding the relevant domain’s developments, impacts, and future directions, highlighting trends, citation networks, and subject clusters in the research (19).

Lately, bibliometric analysis has gained importance in medical research, offering insights into research trends, influential papers, authors, and institutions (20). Bibliometric studies often employ specialized software like VOSviewer, Bibliometrix R Package’s web app Biblioshiny, CiteSpace, etc., for data visualization and mapping (21, 22). In medical sciences, bibliometrics can inform funding decisions, academic promotions, and healthcare planning (23). The growing acceptance of bibliometric analyses in medical literature reflects their value in identifying influential research and guiding future investigations (20).

At present, there is absence of research analyzing the status of palliative care for cervical cancer using the bibliometrics approach. A bibliometric analysis of global research in palliative care for cervical cancer is warranted for several reasons. First, such an analysis can provide a comprehensive overview of the scientific outputs, identify influential journals, authors, and institutions, and highlight the collaboration between countries. Additionally, it can reveal research trends, gaps, and the field’s evolution, which is crucial for guiding future research and policy-making. Interestingly, despite the recognized global health burden of cancer in palliative care and the specific challenges associated with cervical cancer, there is evidence of underutilization of palliative care services (24), suggesting a potential disconnect between research outputs and clinical practice, highlighting the need for bibliometric studies to inform strategies for better integrating palliative care in oncology (14).

The lack of a comprehensive grasp of global research developments and trends in this field underscores the need to bridge this gap. Pinpointing research deficiencies, identifying key contributors, and fostering collaboration are essential to advance palliative care for patients coping with cervical cancer. The current study addresses the critical gap in knowledge by employing a bibliometric analysis to investigate scholarly output on palliative care for cervical cancer thoroughly. This analysis examines publication trends, authorship patterns, influential journals, trending topics, and thematic concentrations within relevant articles to understand the current state of research, identify best practices, and address the unique challenges of managing cervical cancer symptoms. The insights gained from this study are poised to direct future research directions, help develop evidence-based guidelines, and inform healthcare providers and policymakers about the need to enhance palliative care services for individuals affected by cervical cancer.

## 2 Materials and methods

### 2.1 Data collection

The Scopus database was selected as the data source for the current study. Searches were performed on the Scopus database, one of the largest, most comprehensive, and multidisciplinary databases. Scopus includes a plethora of literature within the biomedical and social sciences, encompassing the entire content of Medline (25). To retrieve the available studies, the title, abstract, and keyword search function of Scopus was used with the key terms including (“cervi* cancer” OR “cervical cancer” OR “cervical Neoplasm” OR “cervical Tumor*” OR “cervical Malignant”) AND (“palliative care nurse*” OR “palliative care” OR “hospice” OR “Palliative Treat*” OR “Palliative Therapy” OR “Palliative Supportive Care”). Studies were included after applying the filtration by year (2000-2023), including literature from 01/01/2023 till 31/12/2023 and language (English). The search was conducted on 26^th^ July 2024.

### 2.2 Data analysis

**Figure 1:**
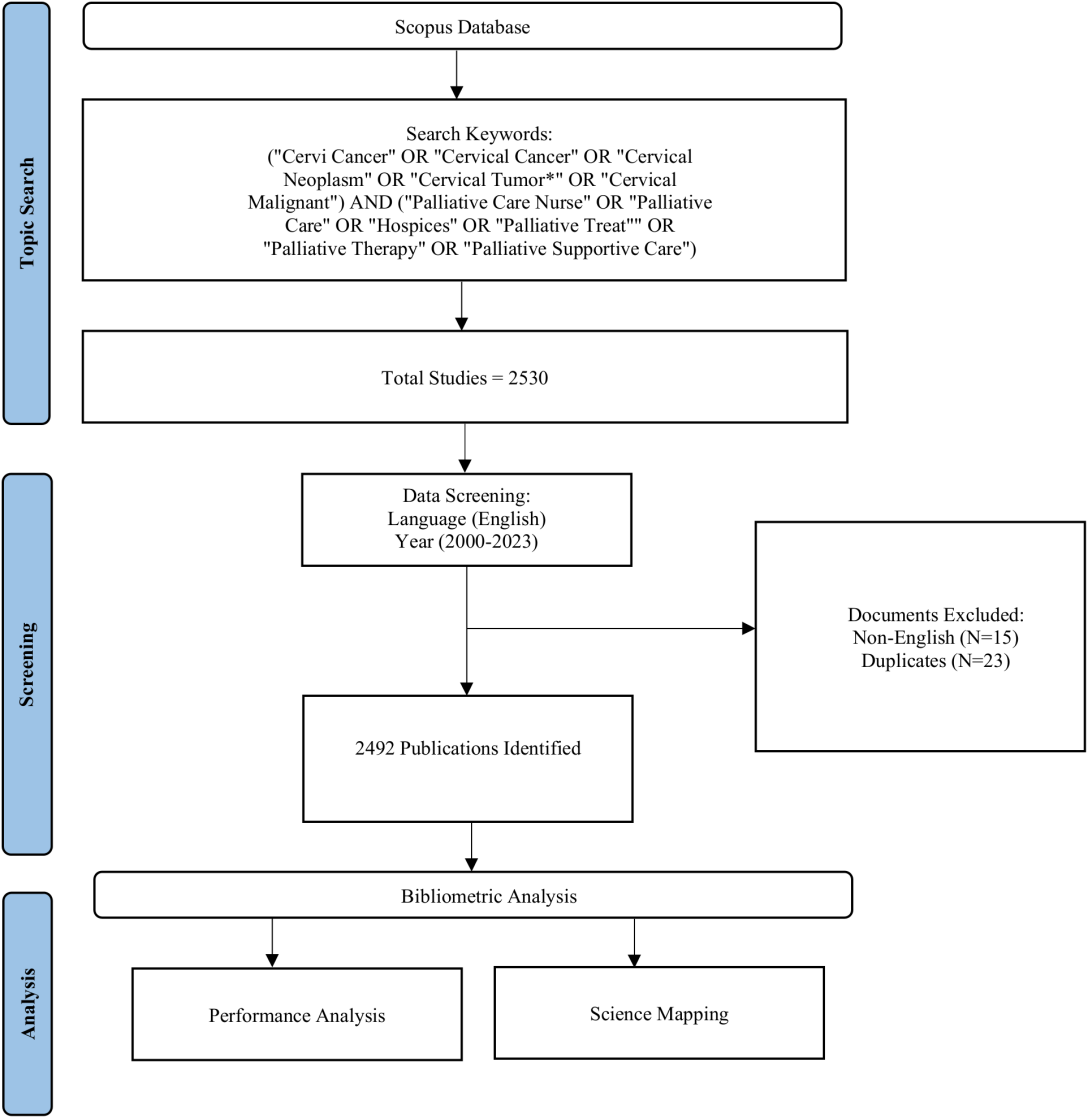
Study flow chart.

After conducting a literature search to attain a comprehensive data set of relevant publications, the next step was to clean and preprocess the data to ensure the accuracy of the dataset and to refine the raw data before analysis. Therefore, all the duplicates and missing entries were removed, and author names, etc., were corrected. Subsequently, the appropriate bibliometric analysis techniques were identified. The two fundamental components of a bibliometric analysis are performance analysis and science mapping. When analyzing research performance, metrics like total publications, author contributions, and citation-related indicators are used to evaluate the influence of researchers, institutions, and nations (26). The structure and dynamics of scientific research can be mapped with science mapping as it helps to gauge the relationships between research constituents. Several techniques are used for science mapping, such as citation and co-citation analysis, bibliographic coupling, co-word analysis, co-authorship analysis, etc. (18, 26). In the current study, performance analysis and science mapping were conducted by employing bibliometric measures such as overall publication trends, contributions of authors, journals, institutions, and countries, productivity and impact analysis of authors and sources/journals, keywords analysis, citation and co-citation analysis, and examining collaboration networks. After selecting the bibliometric techniques and measures, the analysis was performed using suitable software tools. VOSviewer and Bibliometrix R Package’s web app Biblioshiny were used to analyze the data. Lastly, visual representations such as charts, graphs, three-field plots, etc. were created to aid interpretation and the findings were reported concisely. The study flowchart is presented in **Figure 1**. Ethics approval was not required for this study.

## 3 Results

This study identified 2,492 publications about palliative care for cervical cancer published in Scopus between 2000 and 2023, including 79.9% articles and 20.1% reviews. This investigation revealed that 12,804 authors contributed to producing 2,492 manuscripts published in 677 journals and citing 63,994 references.

### 3.1 Trends in publications

**Figure 2:**
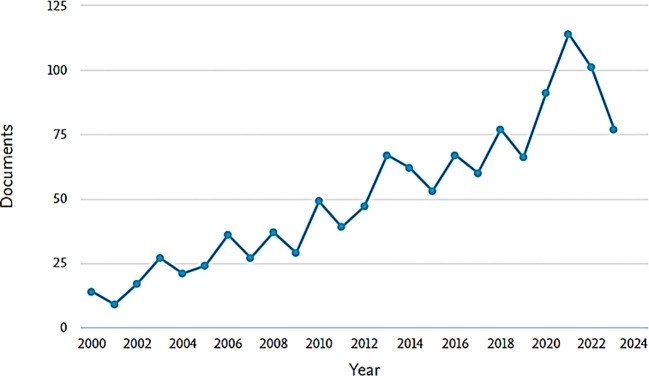
Publications over the years (2000-2023).

The study examined the yearly publication trends to comprehend the evolution of related research. Annual publications during the study period showed a clear upward trajectory (**Figure 2**). Notably, the peak of publications was in 2021 (n=114). Since 2000, when 14 scientific articles were published, the number of publications has steadily increased, although the output was relatively lower in 2023 after peaking in 2021.

### 3.2 Authors analysis

**Figure 3:**
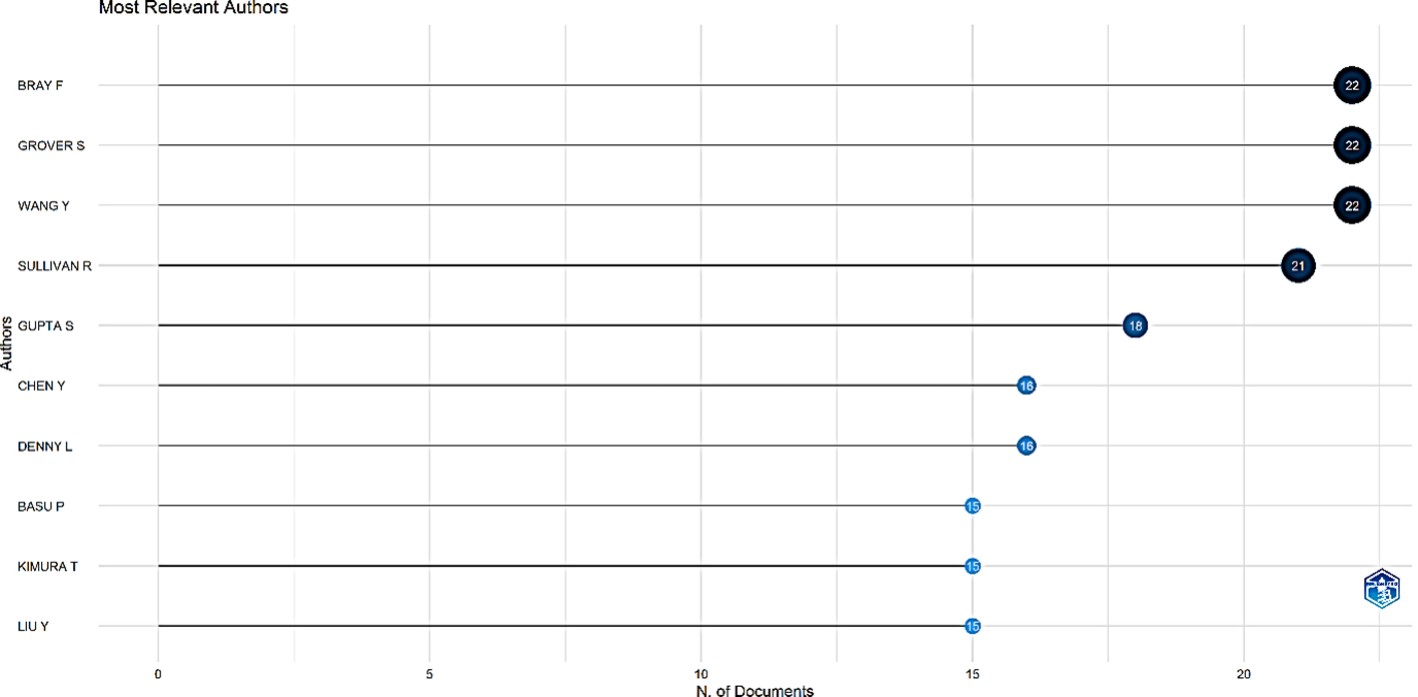
The top 10 most relevant authors.

**Figure 4:**
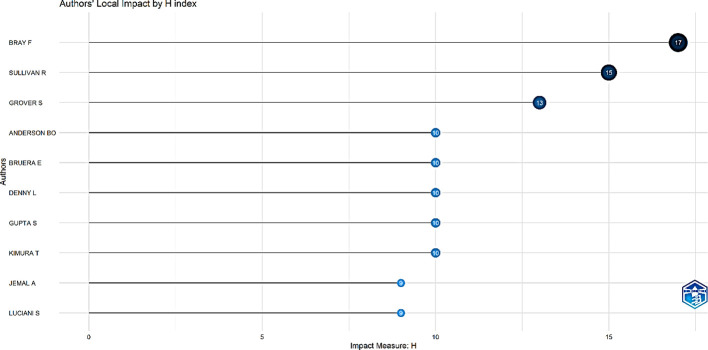
List of top 10 most influential authors by H-index.

Examining the seminal work of impactful authors in a field provides a way to comprehend its classic theories. The H-index is a novel measurement standard for evaluating scholarly achievement. **Figures 3** and **4** showcase the top 10 most relevant authors based on the number of publications and H-index.

A total of 12,804 authors contributed to the 2,492 publications, yielding a co-authorship index of 6.68. There were 119 single-authored documents. **Figure 3** shows the top 10 most relevant authors. Bray F, Grover S, and Wang Y were the most significant authors, with 22 papers each, followed by Sullivan R with 21 papers.

The H-index assists in measuring both the productivity and citation impact of the publications of authors and scholarly journals/sources. The author’s local impact from an H-index perspective indicated that the top three authors were Bray F (H-index=17), Sullivan R (H-index=15), and Grover S (H-index=13).

**Figure 5:**
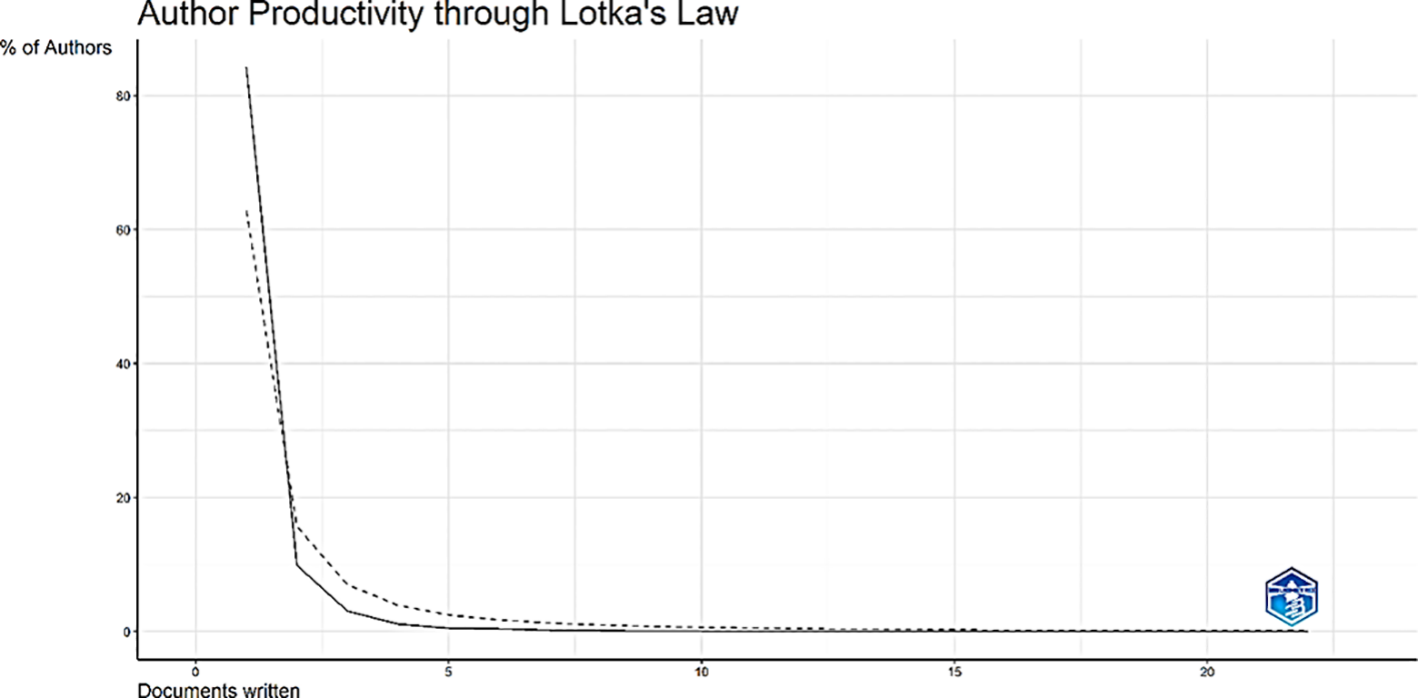
Analysis of author productivity by Lotka’s Law.

Lotka’s law provided a glimpse of the productivity of the authors, revealing that the majority of authors (84.2%) published only one paper, and only 9 (0.1%) authors produced 10 publications (**Figure 5**).

### 3.3 Affiliations/Institutions analysis

**Figure 6:**
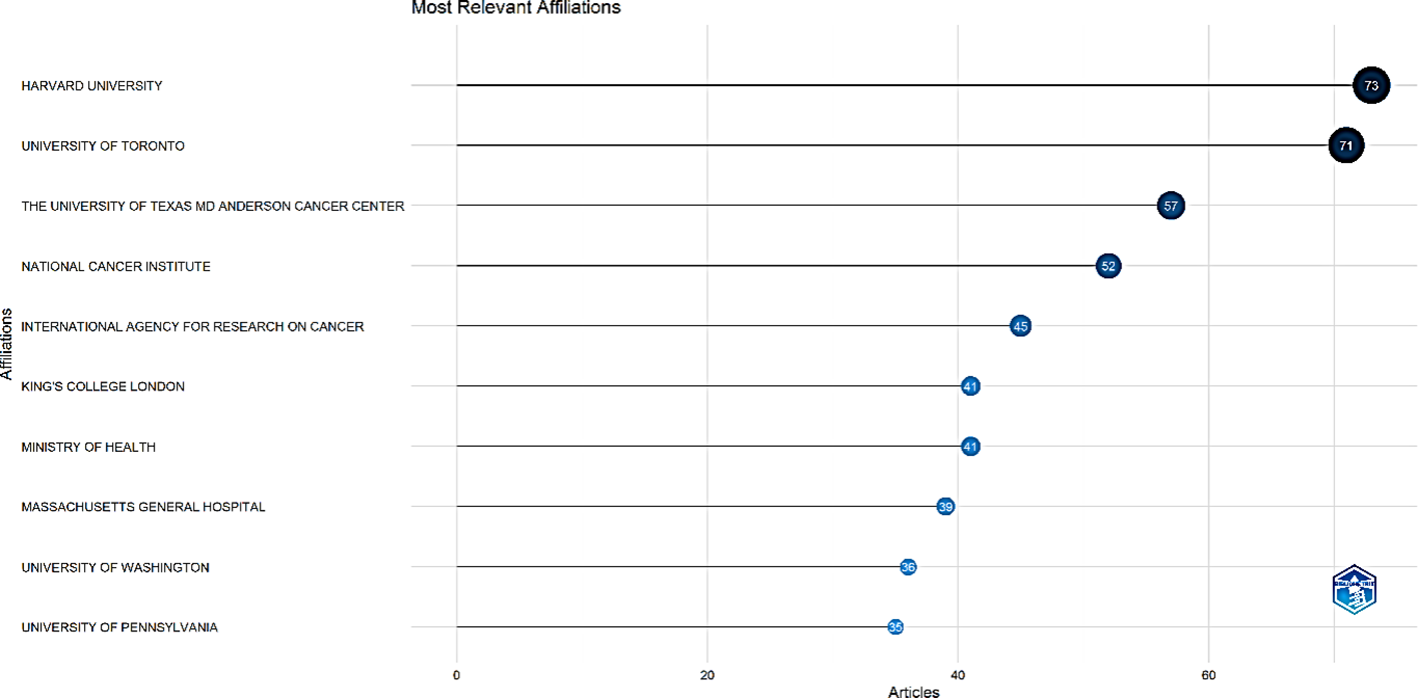
Top 10 most relevant institutions.

Research institutions play a crucial role in advancing research, and the course of top institutions frequently influences the academic direction in the field. Published research on palliative care for cervical cancer originated from a diverse range of 3,885 institutions/affiliations worldwide. Harvard University, the University of Toronto, and the University of Texas MD Anderson Cancer Center were the top three relevant affiliations, with 73, 71, and 57 publications, respectively (**Figure 6**).

### 3.4 Sources/Journals analysis

**Figure 7:**
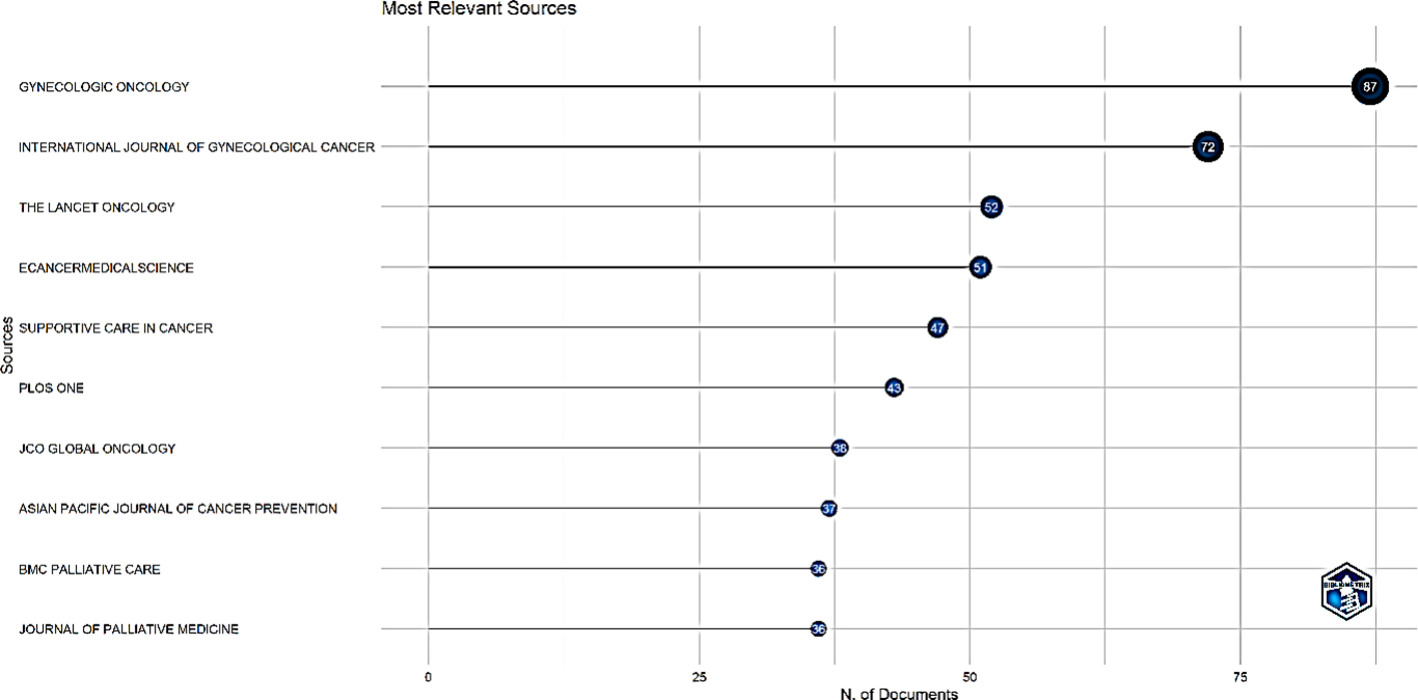
Top 10 most relevant sources.

The identified papers originated from 677 sources, with Gynecologic Oncology being the most relevant source with 87 publications, trailed by the International Journal of Gynecological Cancer with 72 publications (**Figure 7**).

**Figure 8:**
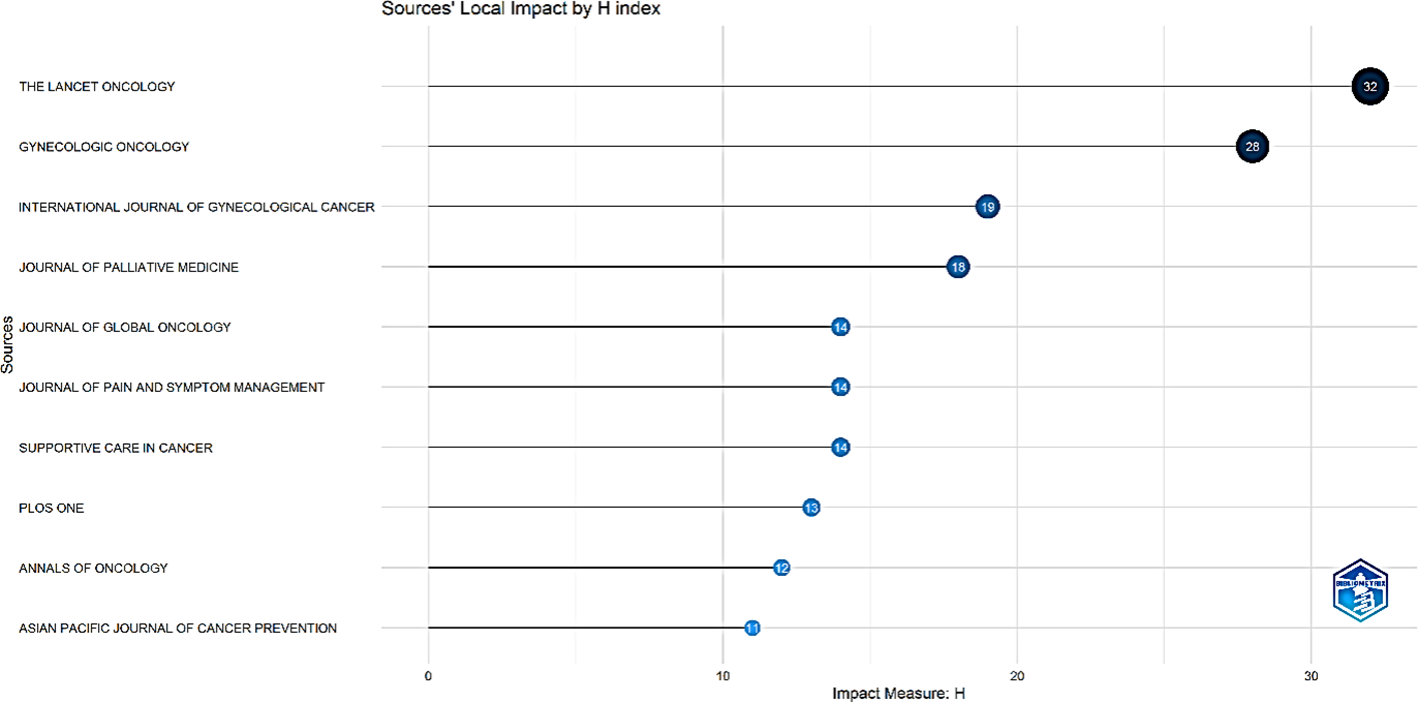
Top 10 influential journals by H-index.

Based on the H-index standard, the top three influential sources/journals in the field of palliative care for cervical cancer were The Lancet Oncology, Gynecologic Oncology, and International Journal of Gynecological Cancer (**Figure 8**).

**Figure 9:**
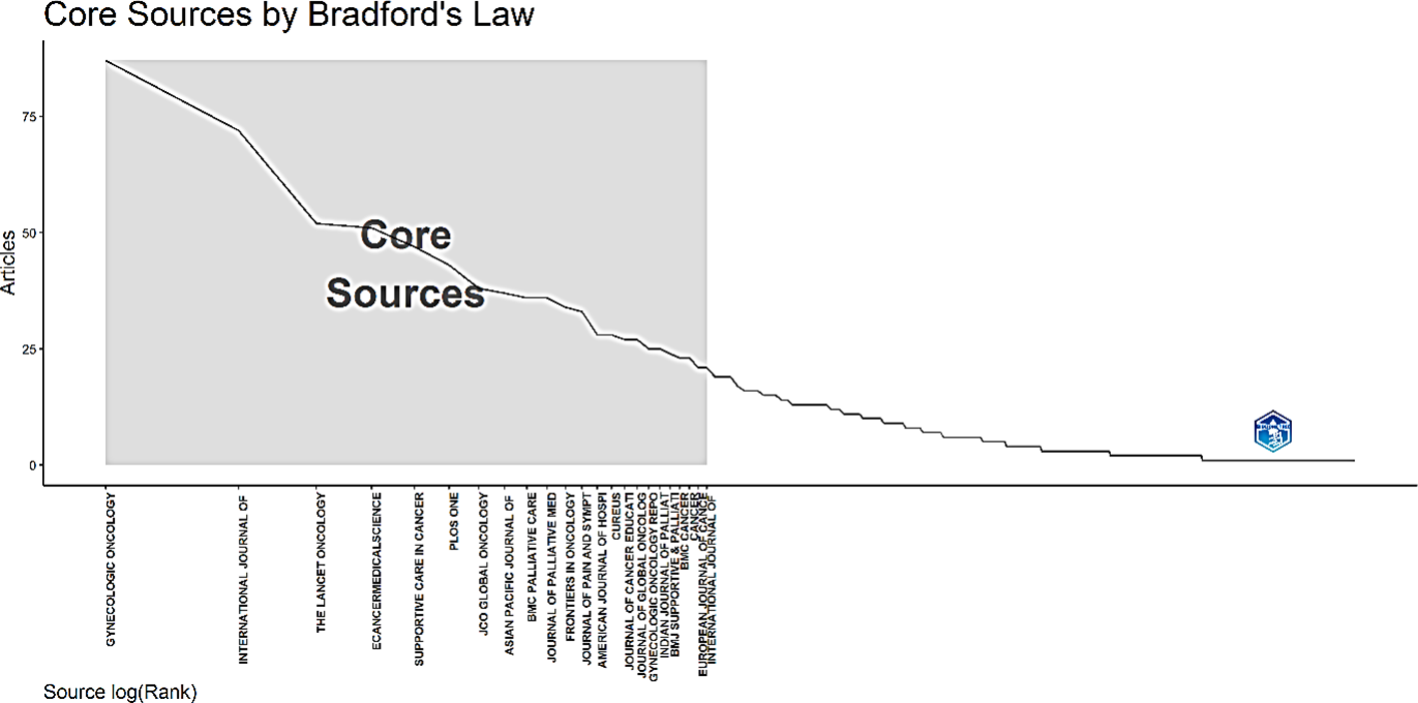
Core sources by Bradford’s Law.

According to Bradford’s law, the core sources of central importance in the domain of palliative care for cervical cancer were as follows: Gynecologic Oncology, International Journal of Gynecological Cancer, The Lancet Oncology, Ecancer Medical Science, Supportive Care in Cancer, PLOS One, JCO Global Oncology, Asian Pacific Journal of Cancer Prevention, BMC Palliative Care, Journal of Palliative Medicine, Frontiers in Oncology, and a few other journals (**Figure 9**).

### 3.5 Countries analysis

**Figure 10:**
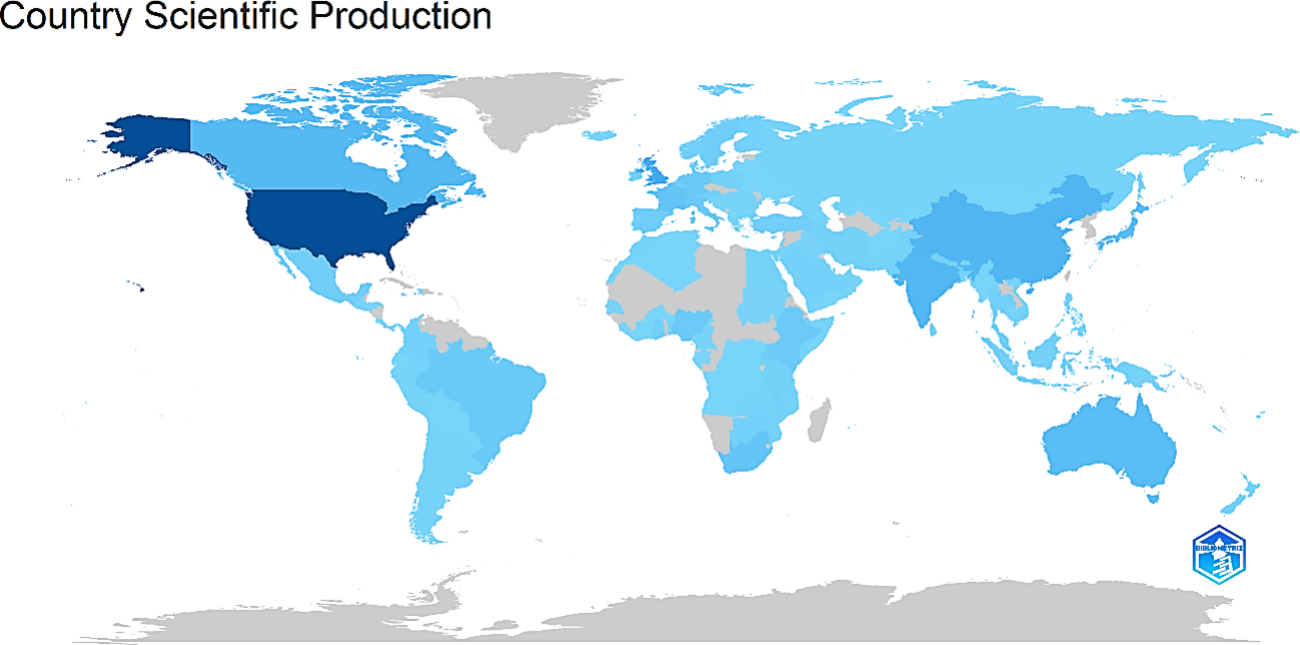
Distribution of publications from different countries/regions.

Publications from 2000 to 2023 were examined in this study to determine which countries have contributed the most to this field of study. The distribution of country-wise scientific production showed the USA having the most studies (827 publications), making up 33.19% of all the documents. UK (257 publications), India (185 publications), and Canada (176 publications) were the subsequent three highest contributors. As shown in **Figure 10**, the countries refer to the locations of the corresponding authors. The depth of the blue color is related to their scientific production: the higher the production, the bluer the color. The USA has the darkest color, indicating the highest scientific production levels.

### 3.6 Citations and co-citations analysis

**Figure 11:**
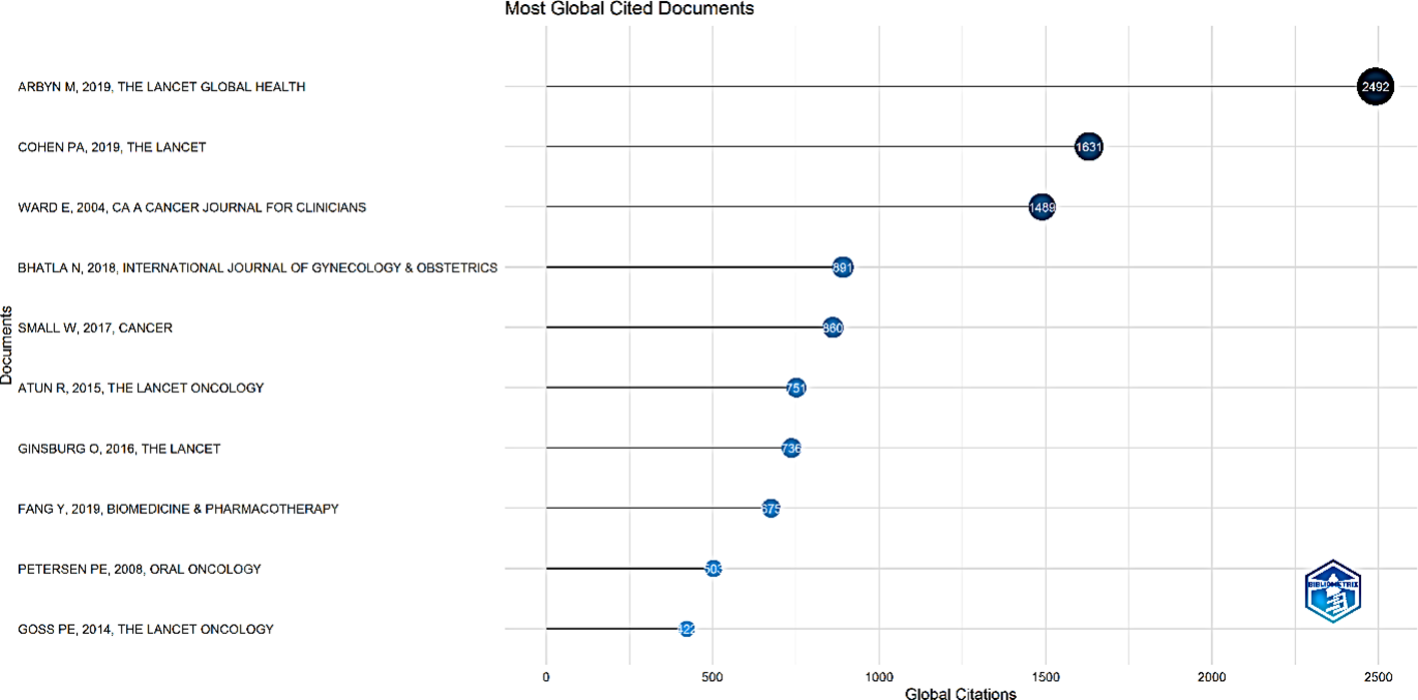
Top 10 most cited documents globally.

Citation analysis was conducted to shed light on the relative importance or impact of publications and countries within the cervical cancer palliative care field using the bibliometric data from 2000 and 2023. Co-citation analysis, in particular, groups papers frequently cited together, allowing researchers to visualize the cognitive structure of a field. Arbyn M, 2019, The Lancet Global Health (Total Citations= 2,492), Cohen Pa, 2019, The Lancet (total Citations=1,631), Ward E, 2004, CA A Cancer Journal for Clinicians (Total Citations=1,489) were the top three most cited publications globally (**Figure 11**).

**Figure 12:**
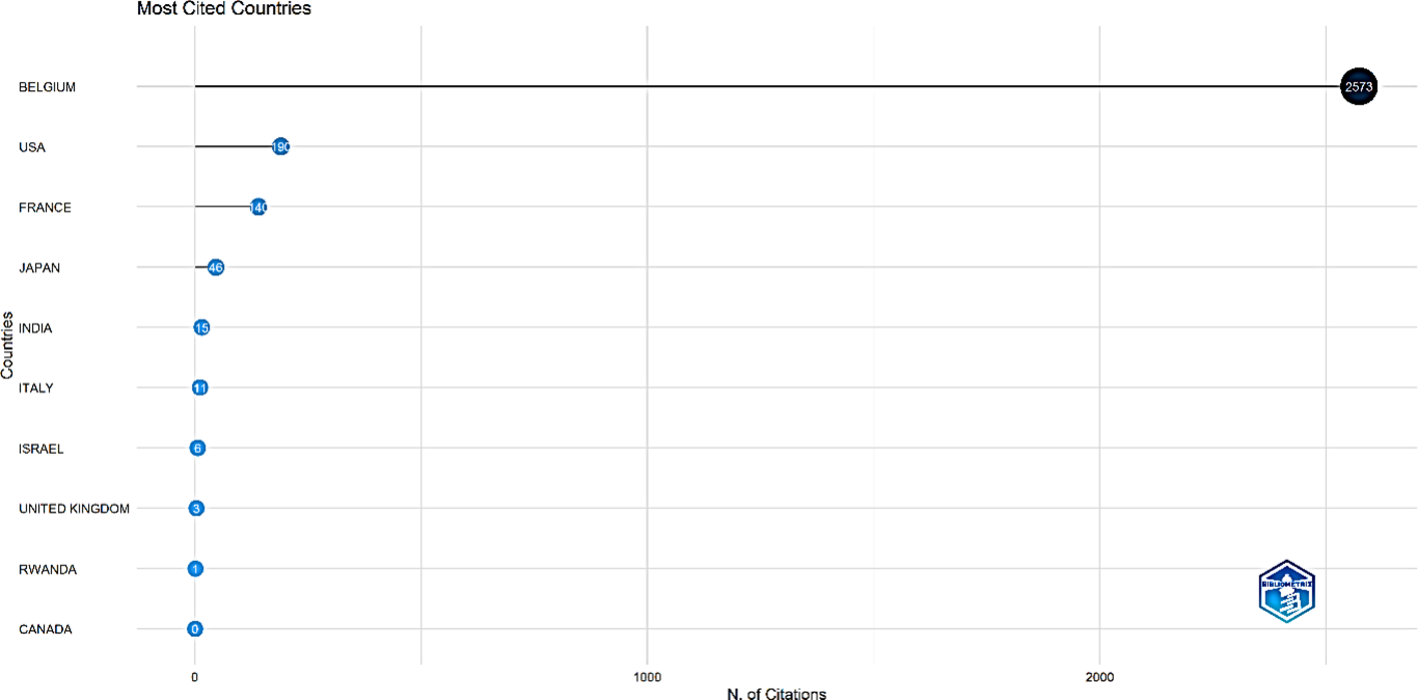
Top 10 most cited countries.

The analysis of the most cited countries revealed some interesting findings. Belgium leads the pack with 2,573 citations, followed by the USA with only 190 citations and France with 140 citations (**Figure 12**).

**Figure 13:**
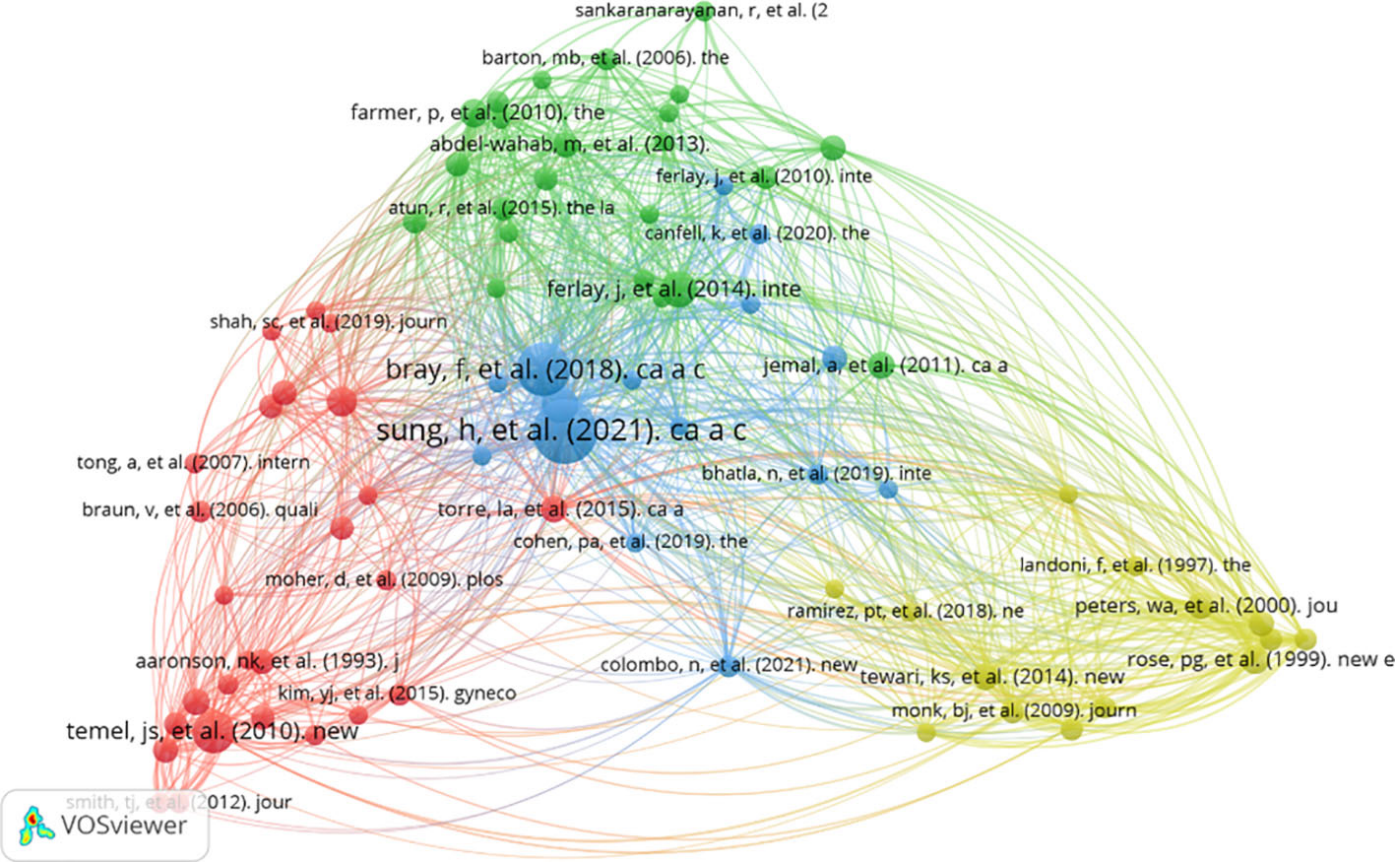
Network visualization map of co-citation of cited documents.

The study carried out a co-citation analysis of the cited references to gain a clear and profound understanding of the structure of the cited references in the palliative care for the cervical cancer field. The analysis obtained a set of 78 references used for co-citation analysis of cited references by applying the threshold 20 times, which indicates that a cited reference should have a minimum of 20 citations. The result showed that the 78 reference sets are divided into four clusters; each color represents a cluster. Bray F (2018), Sung H (2021), Temel JS (2010), and Ferlay J (2014) held the most co-citation collaboration/relationship networks (**Figure 13**).

### 3.7 Keywords analysis

**Figure 14:**
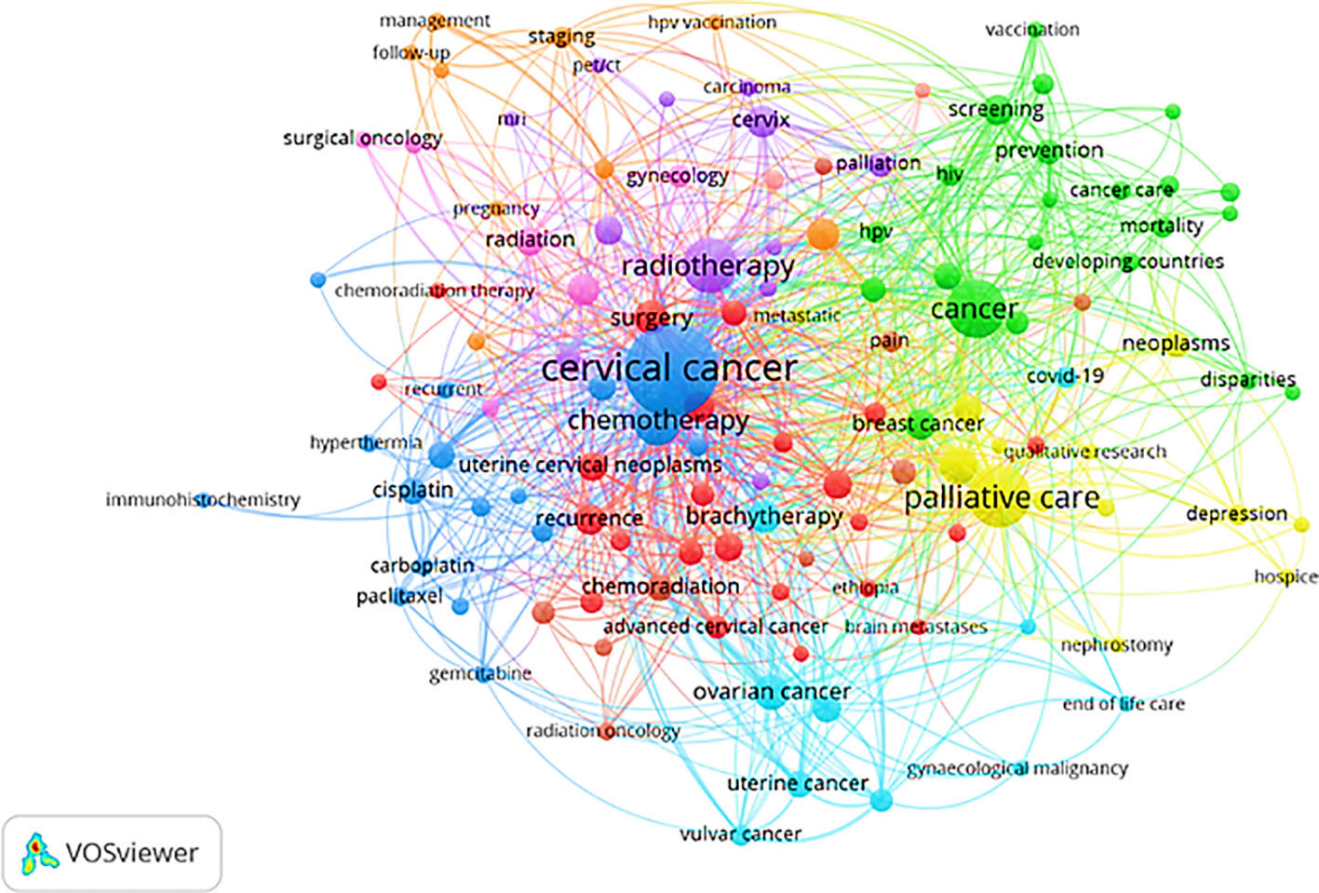
Network visualization map based on author’s keywords.

Keywords are crucial indicators of the central themes of a publication, and keyword analysis helps reveal the main focus areas, patterns of trends, and thematic paths within a field. An author’s keyword analysis is presented in **Figure 14**. It was revealed that the prevalent author keywords linked to cervical cancer palliative care included “cervical cancer”, “palliative care”, “radiotherapy”, “chemotherapy”, “quality of life”, “ovarian cancer”, “brachytherapy”, and “quality of life (pro)/palliative care”, highlighting the research hotspots. The analysis identified 9 clusters, and the most prominent 3 clusters are colored dark blue, green, and yellow. Based on synthetic knowledge synthesis (SKS), a triangulation of bibliometrics, bibliometric mapping, and content analysis, each cluster can be categorized into themes (27). The dark blue colored cluster consisted of keywords “cervical cancer”, “uterine cervical neoplasms”, “chemotherapy”, “gemcitabine”, “cisplatin”, and “carboplatin”. Hence, the identified theme of the dark blue cluster was cervical cancer and its treatments. The keywords that formed the yellow cluster were: “palliative care”, “depression”, “hospice”, “nephrostomy”, “neoplasms”, and “qualitative research”. The theme of the yellow cluster revolved around palliative care and its measures. The green cluster comprised keywords “screening”, “prevention”, “vaccination”, “HPV”, “HIV”, “cancer”, “mortality”, and “developing countries”. The theme of the green cluster highlighted inoculation and early detection of cervical cancer.

**Figure 15:**
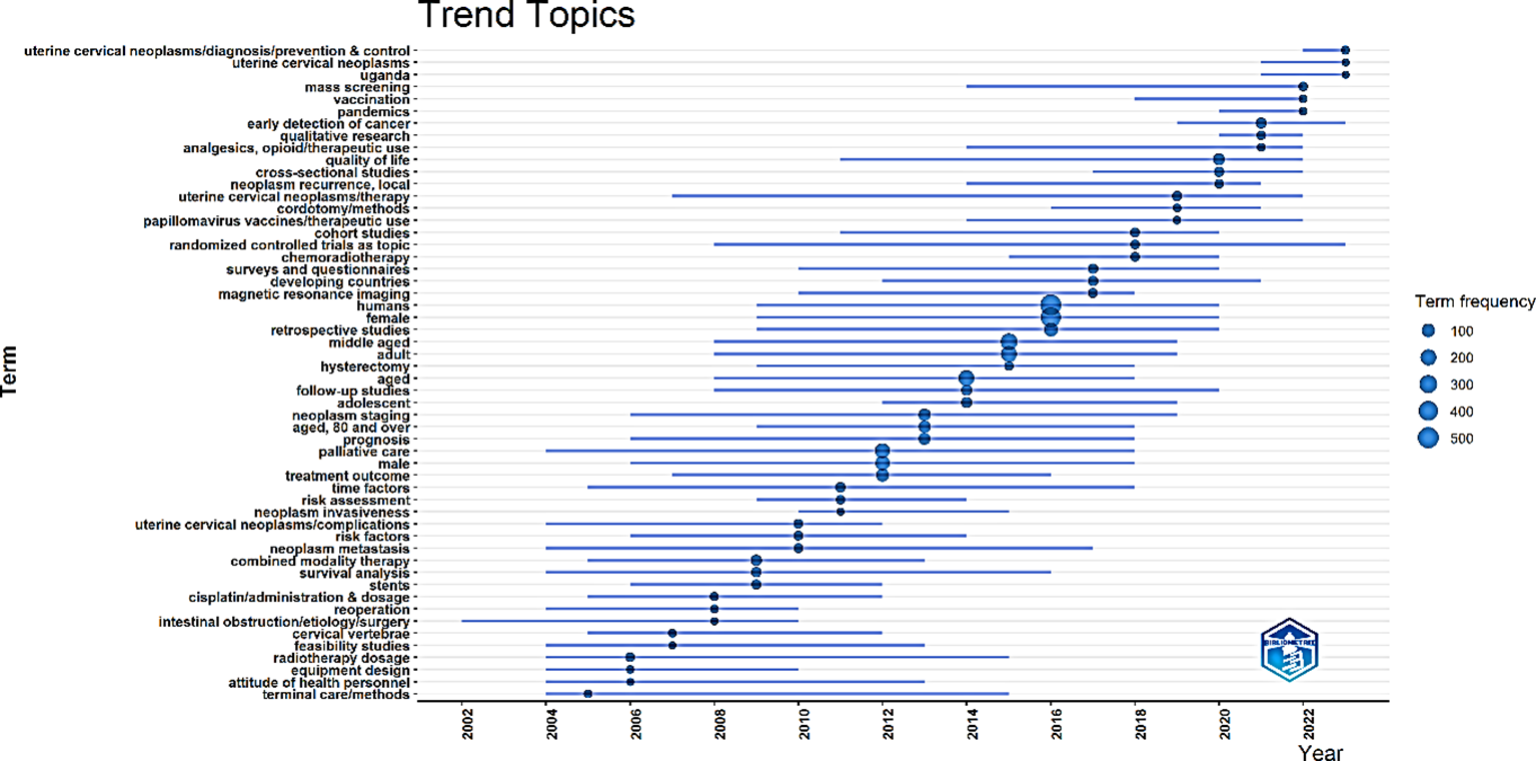
Trend topics from 2000-2023.

**Figure 15** illustrates the changing trend of the most popular keywords related to palliative care for cervical cancer over time. The trend of the occurrence of the most commonly used words for the topic from 2000 to 2023 is shown in this figure. The size of the circles represents the words’ frequency of occurrence, and the length of the lines indicates their duration. The most popular words, research hotspots, or trend topics observed from 2020 onwards were uterine cervical neoplasms/diagnosis/prevention & control, qualitative research, etc.

### 3.8 Collaboration networks analysis

**Figure 16:**
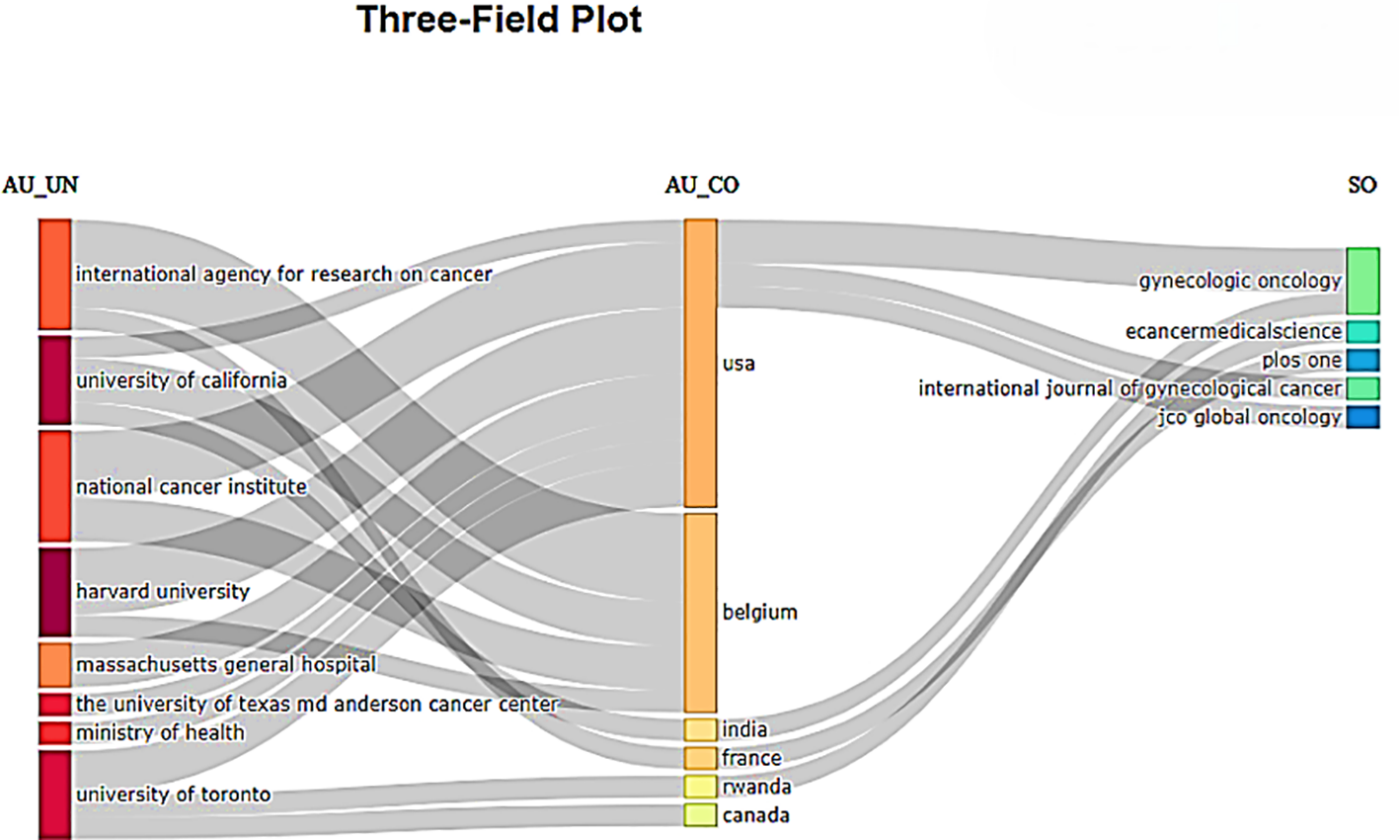
Three-field plot showing the network between institutions (left), countries (middle), and journals (right).

Collaboration network analysis determines relationships between bibliographic elements like authors and institutions, manifesting scientific collaboration. It employs social network analysis techniques to examine cooperative behaviors between authors, institutions, journals, and countries. A three-field plot, also known as a Sankey chart, is displayed in **Figure 16** and illustrates the relationships between sources, affiliations, and countries. The height of the rectangular nodes in the collaborative network is directly proportional to the frequency of the presence of a nation, affiliation/institution, or source/journal. The number of connections is directly proportional to the width of the lines connecting the nodes. The results showed that the USA was the dominant country, comprising most of the top 10 institutions, followed by Belgium. In the source/journal section on the right, USA was the leading contributor to Gynecologic Oncology.

**Figure 17:**
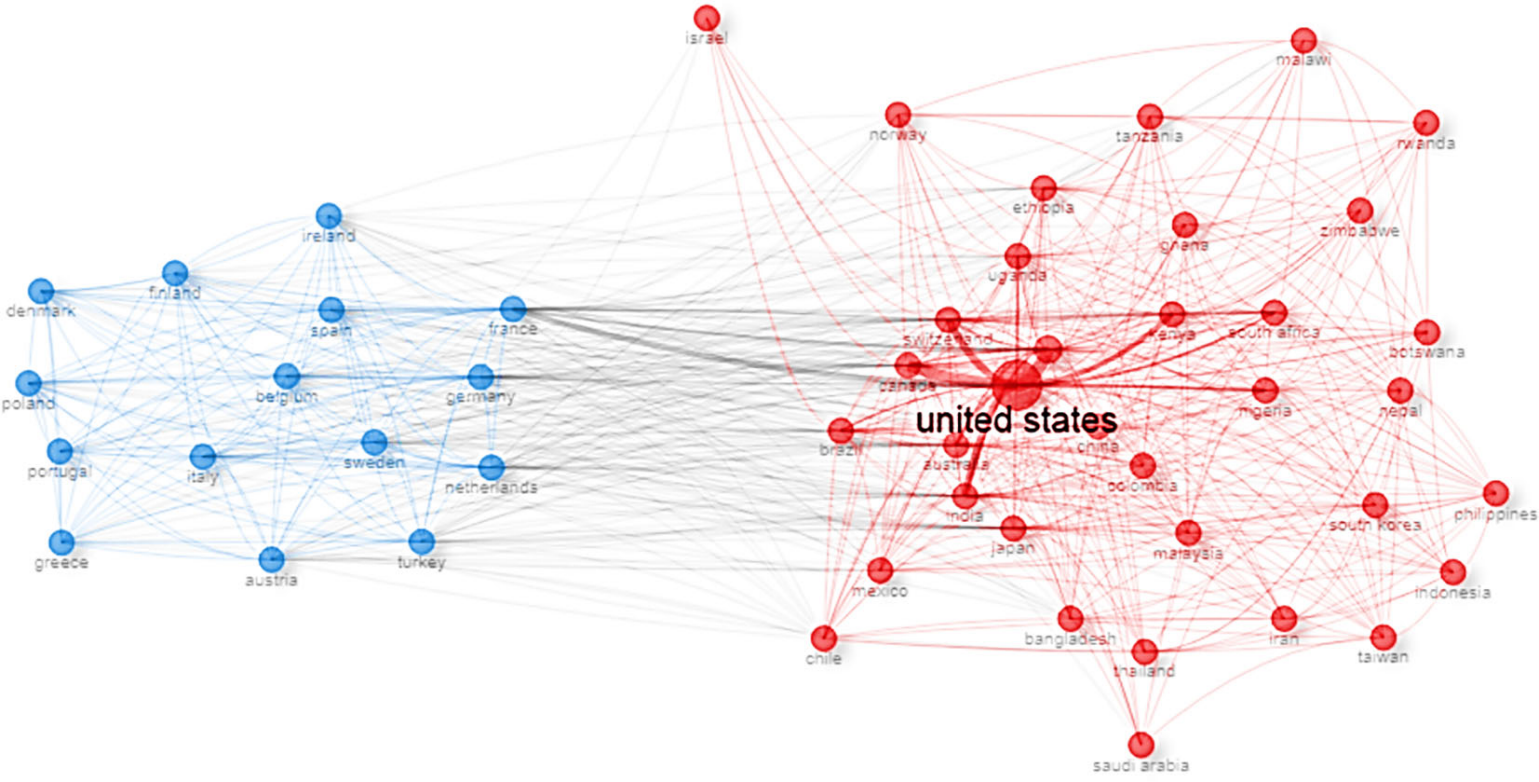
The collaboration network between countries.

Co-authorship among countries measures the cooperative associations based on the number of co-authored documents. **Figure 17** portrays the collaboration network of the most productive countries. It presented that the USA maintains close relationships with other countries and has a vast collaborative bond with numerous countries.

## 4 Discussion

The current study conducted a scientometric analysis of 2,492 documents on palliative care for cervical cancer published from 2000 to 2023. Analysis unveiled that during this period, there was a steady increase in the overall quantity of scientific output. The production of articles grew at an average annual rate of 10.82% and peaked in 2021. Although 2021 is well noticed for the COVID-19 pandemic lockdown, studies have shown that the COVID-19 pandemic has led to a significant increase in research publications in 2021 (28). The upward trend indicated that the relevant research field thrived from 2000 to 2023. Notably, the rise in the number of publications on palliative care for cervical cancer can be attributed to the increase in the incidence and mortality of cervical cancer, as well as the progression in awareness about the effective role of palliative care in symptom management. In the future, with the introduction of additional guidelines and the maturation of palliative care models, one can expect a continued expansion of literature in this field. Moreover, the high average citations per article implied that publishing research on palliative care for cervical cancer in reputable journals poses no challenge.

12,804 prolific authors contributed towards the research area of interest. The authors’ analysis used the number and citations of publications to assess individual authors. Bray F emerged as the most significant author regarding the total number of publications and H-index. He also secured the most co-citation collaborative networks. The analysis of countries/regions discovered North America (USA and Canada), the UK, and India as the hubs for scientific production in this field. Notably, with 827 papers, the United States distinguished itself as the top nation in scientific output, but citation-wise, Belgium aced the contest with 2,573 citations. Based on the total number of publications, the US was at the forefront of this field’s research, and surprisingly, Belgium led in the citation count. The country-wise contribution to palliative care for cervical cancer publications was dominated by high-income countries, indicating that publications on this subject are predominantly centred in developed countries, highlighting a significant imbalance in research infrastructure and funding, leaving lower-income regions behind in this crucial field.

3,885 institutions and 677 journals were identified as the providers of publications in palliative care for cervical cancer. Harvard University appeared as the most productive institution in the field, while the Gynecologic Oncology firmed its place as the most relevant journal. However, the H-index metric ranked Lancet Oncology as the most influential journal. The collaboration network analysis depicted that the most prominent cluster was comprised primarily of USA-based institutions. Also, the USA contributed the most to the most pertinent journal, Gynecologic Oncology. The co-authorship collaborative ventures based on country showed that the US authors actively collaborated with a vast network of authors from other countries. Therefore, broadening collaborative networks to encompass additional geographic areas to support developing countries is advisable.

The authors’ keyword examination gave insight into the central themes and trends in publications related to palliative care for cervical cancer, indicating an increased research emphasis on vaccination and early diagnosis, cervical cancer treatment, and enhancement of cervical cancer care by integrating palliative care into standard clinical management. The thematic research areas in palliative care for cervical cancer included surgery and survival, cervical cancer, palliative care and hospice, palliative chemotherapy, radiotherapy, ovarian cancer, treatment and diagnosis, cancer pain, quality of life (pro)/palliative care, screening, and prevention. The areas were found to be consistent with the subject domain theme. The growing attention to palliative care in cervical cancer is promising yet underexplored. It is pertinent to explore the role of palliative care in managing physical, social, spiritual, and psychological distress among cervical cancer patients, highlighting the need for dedicated research efforts to explore this potential synergy.

## 5 Limitations

The current research’s analysis was conducted on data retrieved from Scopus, which offers extensive coverage of peer-reviewed literature but may not contain all journal publications across all disciplines. This decision was justified by software limitations and the inherent challenges associated with merging data from diverse databases. Exclusion criteria further imposed a language restriction, limiting the study to English-language publications, which inevitably omitted some potentially relevant articles. Moreover, as with any bibliometric analysis, the chosen keywords and search string might have inadvertently excluded relevant studies. No search strategy can capture all pertinent research, and alternative approaches could reveal further nuances within the field. Therefore, while this analysis offers valuable insights into the global landscape of palliative care for cervical cancer research, future research can build upon it by addressing these limitations and expanding the scope of inquiry to optimize palliative care strategies further and improve patient outcomes worldwide.

## 6 Conclusion

The present study employed a bibliometric approach to explore the current research landscape and emerging trends in palliative care for cervical cancer, focusing on publications indexed in Scopus between 2000 and 2023. Based on the findings, it was evident that there has been a steady increase in the number of publications on this topic over the past two decades. However, the study unveils limited collaborative engagement among nations and intercountry institutional networks, with research predominantly concentrated in high-income economies. As such, there is a notable scarcity of studies conducted in developing countries. Therefore, to facilitate the widespread adoption of palliative care services in cervical cancer treatment worldwide, aggressive collaborative efforts are required. Such as enhancing global and local collaboration in research, augmenting funding assistance, and conducting cutting-edge research that addresses the specific needs and demands of different regions. By fostering cooperation and conducting demand-driven research, advancements in the realm of palliative care for cervical cancer can be achieved, ultimately improving the quality of care for patients globally (29).
